# Searching for “monogenic diabetes” in dogs using a candidate gene approach

**DOI:** 10.1186/2052-6687-1-8

**Published:** 2014-07-07

**Authors:** Andrea D Short, Angela Holder, Simon Rothwell, Jonathan Massey, Rachel Scholey, Lorna J Kennedy, Brian Catchpole, William ER Ollier

**Affiliations:** Centre for Integrated Genomic Medical Research, University of Manchester, Stopford Building, Oxford Road, Manchester, M13 9PT UK; Department of Pathology and Pathogen Biology, Royal Veterinary College, Hawkshead Lane, North Mymms, Hatfield, Herts AL9 7TA UK

**Keywords:** Diabetes mellitus, Dog breeds, Candidate genes

## Abstract

**Background:**

Canine diabetes is a common endocrine disorder with an estimated breed-related prevalence ranging from 0.005% to 1.5% in pet dogs. Increased prevalence in some breeds suggests that diabetes in dogs is influenced by genetic factors and similarities between canine and human diabetes phenotypes suggest that the same genes might be associated with disease susceptibility in both species. Between 1-5% of human diabetes cases result from mutations in a single gene, including maturity onset diabetes of the adult (MODY) and neonatal diabetes mellitus (NDM). It is not clear whether monogenic forms of diabetes exist within some dog breeds. Identification of forms of canine monogenic diabetes could help to resolve the heterogeneity of the condition and lead to development of breed-specific genetic tests for diabetes susceptibility.

**Results:**

Seventeen dog breeds were screened for single nucleotide polymorphisms (SNPs) in eighteen genes that have been associated with human MODY/NDM. Six SNP associations were found from five genes, with one gene (*ZFP57*) being associated in two different breeds.

**Conclusions:**

Some of the genes that have been associated with susceptibility to MODY and NDM in humans appear to also be associated with canine diabetes, although the limited number of associations identified in this study indicates canine diabetes is a heterogeneous condition and is most likely to be a polygenic trait in most dog breeds.

**Electronic supplementary material:**

The online version of this article (doi:10.1186/2052-6687-1-8) contains supplementary material, which is available to authorized users.

## Lay summary

Diabetes is a common condition where sugar (glucose) levels of the body are poorly regulated, due to either lack of production of the hormone insulin, made in the pancreas, or an increase in resistance of tissues in the body to the effects of insulin. Canine diabetes is similar to some forms of human diabetes; it is relatively common in dogs, and its prevalence (the proportion of dogs affected at a point in time) ranges from 0.005% to 1.5%, and is dependent on which breed is being considered. This breed-related variation in the prevalence of diabetes suggests a genetic basis in dogs.

In humans, some forms of diabetes are due to mutations in just a single gene (these are called monogenic conditions). This study examined 18 genes that are known to be associated with human monogenic diabetes, and tested whether they are also associated with diabetes in 17 different dog breeds.

Six variants from five genes were found to be associated with diabetes in some breeds. Interestingly, two different variants in the same gene (called ZFP57 - Zinc finger protein 57) were associated with diabetes in two different breeds.

## Background

Canine diabetes is a common endocrine disorder with an estimated prevalence ranging from 0.005% to 1.5% [1]. Almost all diabetic dogs require exogenous insulin therapy to manage their hyperglycaemia, often resulting from insulin deficiency leading to the inability to control their blood glucose concentration. Canine diabetes has been compared with human type 1 diabetes (T1D) [2, 3] as they share many clinical and pathophysiological features. However, in contrast with T1D, which is usually diagnosed in young patients (<30 years of age), canine insulin-deficiency diabetes (IDD) occurs more commonly in older dogs, aged 7–12 years [4].

The aetiology and underlying pathogenesis of canine IDD has not been fully determined, although exocrine pancreatic disease [5, 6] and immune-mediated mechanisms [7] are suspected to be underlying causes of pancreatic beta cell destruction. It has been also been suggested that, in many ways, canine diabetes resembles latent autoimmune diabetes of the adult (LADA) in man [8], a more slowly progressive form of autoimmune diabetes.

Pedigree dog breeds, similar to some ethnic groups in the human population [9], display variability in diabetes susceptibility, with some breeds (e.g. Samoyed) being over-represented, whereas others (e.g. Boxer) seem to be relatively resistant to developing the disease [10]. These breed-related differences in diabetes susceptibility suggest that the pathogenesis of diabetes is influenced by genetic factors and similarities between canine and human diabetes phenotypes indicate that the same genes and/or genetic pathways might be involved in both species. Since some phenotypes also appear to be somewhat breed-specific [11], for example NDM, in Keeshond dogs [12] and dioestrus diabetes in female entire Elkhounds and Lapphunds [13], there could be differences in the individual susceptibility genes that contribute to the overall genetic risk for different dog breeds, as is seen with different ethnic groups and type 2 diabetes in humans [14].

A small proportion of human diabetic patients suffer from disease resulting from mutation(s) in a single gene. These monogenic forms of diabetes account for around 1-5% of human diabetes cases and include maturity onset diabetes of the young (MODY) and neonatal diabetes mellitus (NDM) [15]. MODY represents a heterogeneous group of disorders that are commonly diagnosed before 25 years of age in humans. They result from autosomal dominant mutations in genes that control the synthesis or secretion of insulin by the pancreatic beta cells and include *HNF4A* (MODY1) [16], *GCK* (MODY2) [17, 18], *HNF1A* (MODY3) [19, 20], *PDX1* (MODY4) [21] and *HNF1B* (MODY5) [22]. NDM is commonly diagnosed around 6 months of age in humans and can be the result of sporadic or inherited (autosomal dominant) mutations in certain genes, including *KCNJ11*, *ABCC8* and insulin (*INS*) [23, 24]. Mutations in the glucokinase (*GCK*) gene can also lead to NDM [25].

Although the majority of diabetic dogs (>90%) are diagnosed in animals over 6 years of age [26], within the population of young diabetic dogs (diagnosed <6 years), there is a clear breed-related overrepresentation of Golden and Labrador Retrievers [26]. Screening for mutations in canine *KCNJ11* and *INS* have not so far identified any genetic anomalies in a cohort of dogs in the United Kingdom that were affected with NDM (Catchpole unpublished data). NDM has been reported in a small number of dog breeds in the USA [27], including an inherited form in Keeshonds, where the specific genetic defect was not identified, but which is believed to be inherited in an autosomal recessive manner [12].

While the domestication of the dog from the wolf is believed to have occurred some 15,000-200,000 years ago [28], most modern pedigree dog breeds have been created in the last 300 years and represent distinct, genetically segregated populations with high levels of inbreeding and reduced heterogeneity. The relatively short time frame taken to establish modern breeds has been insufficient for chromosomal restructuring to take place and as a result of this, they have extended linkage disequilibrium (LD) and long haplotype blocks within a breed [29]. The selection bias for ‘desirable’ morphological and behavioural traits (hunting instinct, head shape etc.) that has been used to create modern breeds has resulted in a concentration of the gene(s) associated with the trait within a given breed. Inadvertently, disease-associated genes have also been concentrated alongside the morphological and behavioural traits, resulting in each breed demonstrating highly variable disease incidences for particular conditions [30, 31].

Identification of a monogenic type of diabetes in a particular dog breed could lead to the development of a breed-specific genetic test for diabetes susceptibility.

To date, the genes that have been identified as causing monogenic types of diabetes in humans have not been evaluated in the diabetic dog population, where it is possible that some breeds may express a monogenic form of the condition. The aim of the present study was to screen single nucleotide polymorphisms (SNPs) from eighteen genes that have been associated with human MODY/NDM in seventeen dog breeds in order to resolve, at least in part, canine diabetes breed-related genetic susceptibilities. Although samples sizes were relatively small for some breeds examined it has been recognised that only 20–50 affected dogs are usually required for identifying conditions with a monogenic aetiology [32].

## Results

### Data quality

Twelve of the 65 genotyped SNPs were excluded prior to analysis because of high failure rates or improper clustering, leaving 53 SNPs for analysis in each breed. Excluded assays were: rs24533550 (Cel), rs22588616, rs9179252 (EIF2AK3), rs22686866 (INS), rs22686870 (INS) rs8516455 (KCNJ11), rs8516454 (KCNJ11), rs8971148 (PAX4), rs9089163 (PDX1), rs21958943 (RFX6), rs22261809 (WFS1), rs23916066 (ZFP7). The final number of SNPs that passed QC for each breed is shown in Table 1, in addition to the total number of cases and controls for each breed that were genotyped and that the number that subsequently passed QC.

**Table 1:** **Breeds used in the study and overview of SNP analysis**

	Case (n)		Control (n)		SNPs (n)
Breed	Genotyped	After QC	Genotyped	After QC	After QC
Bichon Frise	29	28	29	29	47
Border collie	82	76	82	79	49
Border terrier	26	24	28	25	50
Cairn terrier	45	42	45	39	52
Cavalier King Charles Spaniel	52	50	55	46	51
Cocker Spaniel	63	58	66	66	44
Doberman	18	17	19	18	51
Jack Russell terrier	61	58	60	54	45
Labrador Retriever	153	136	155	155	46
Miniature Dachshund	39	37	40	39	47
Miniature Schnauzer	32	29	31	29	49
Samoyed	41	40	84	74	50
Springer Spaniel	22	21	25	25	48
Staffordshire Bull terrier	15	14	15	15	52
Tibetan terrier	30	30	30	30	51
West Highland White terrier	135	123	135	129	45
Yorkshire terrier	79	76	80	75	47
Total	922	859	979	927	

### Allele association

Allelic analysis identified six SNPs associated with canine diabetes in this study (Table 2). *ZFP57* was associated with canine diabetes in two different breeds, although the associated marker was different: Bichon Frise (rs23901704) and Samoyed (rs23892119) (Table 2). Diabetes in Cocker Spaniels showed an association with three SNPs from three different genes: *MTTL1* (rs24305581), *PAX4* (rs22302353) and *INS* (rs22686871) and in the miniature Dachshund there was a single association with *HNF4A* (rs8804236).

**Table 2:** **Allelic associations**

					Minor/associated allele	Frequency		Major allele	***p***-values			Odds ratio	
	Gene	CFA	Position (bp)	Annotation		Case	Control		Raw	Bonferroni	Permutation		95% CI
Bichon Frise													
rs23901704	ZFP57	35	26,267,827	Syn. coding	T	0.00	0.24	C	2.58E-04	7.21E-03	4.30E-03	nc	nc
Cocker Spaniel													
rs24305581	MTTL1	5	82,755,249	Intronic	A	0.44	0.23	G	3.73E-04	1.05E-02	1.13E-02	2.67	1.54-4.61
rs22302353	PAX4	14	8,718,207	Intronic	A	0.09	0.33	G	6.45E-06	1.81E-04	3.00E-04	0.21	0.10-0.43
rs22686871	INS	18	46,324,586	Syn. coding	G	0.00	0.13	T	7.22E-05	2.02E-03	1.90E-03	nc	nc
Miniature Dachshund													
rs8804236	HNF4A	24	31,897,491	Syn. coding	G	0.14	0.36	A	1.45E-03	4.62E-02	4.31E-02	0.28	0.12-0.63
Samoyed													
rs23892119	ZFP57	35	26,270,002	Intronic	C	0.11	0.00	T	1.55E-04	5.10E-03	5.50E-03	nc	nc

CFA = canine chromosome number: Permutations: n=10,000: CI = confidence interval: Syn. Coding = synonymous coding SNP; nc = not calculable as one of the populations does not carry one of the alleles. Allele associations are calculated using the minor allele as a reference.

Of the associated SNPs, three were intronic (rs24305581, rs22302353, rs23892119) and three were synonymous coding SNPs, (rs23901704, rs22686871 rs8804236, Table 2).

### Genotype association

**Figure 1:**
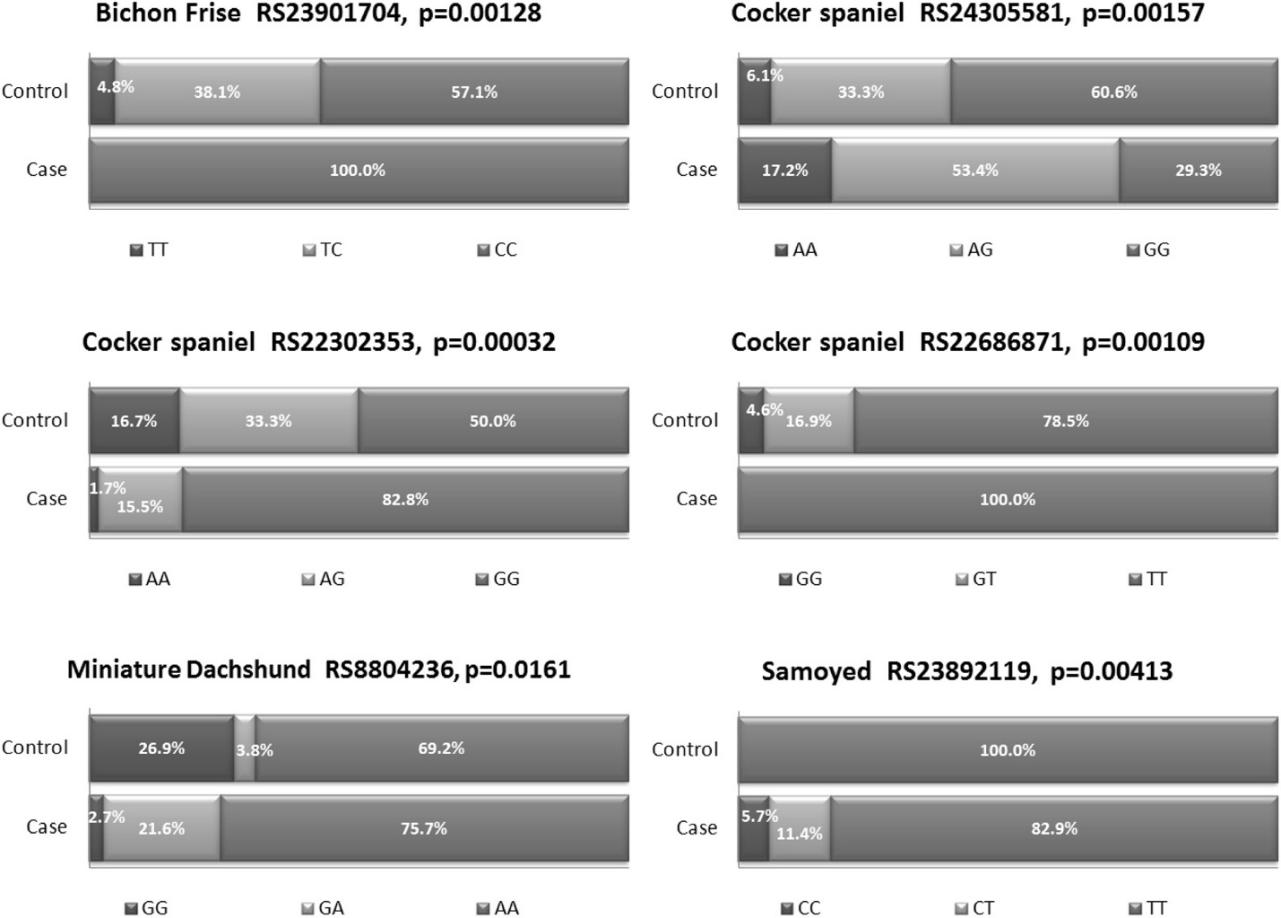
**Genotype distribution of associated SNPs in diabetic cases and controls of specified breeds.**

Genotype analysis revealed a significant association with all of the SNPs identified above (*p*<0.05, Figure 1). In the Bichon Frise, the ‘T’ allele of SNP rs23901704 was associated with reduced risk for diabetes, with the TT and TC genotypes being found in 4.8% and 38.1% of the controls respectively. These two genotypes were not found in any of the cases, where all of the diabetic dogs of this breed carried the CC genotype compared to 57.1% of the controls (Figure 1). In the Samoyed, the ‘C’ allele of SNP rs23892119 was associated with increased risk for diabetes and represented 11% of the case population alleles; this allele was not found in the controls (Table 2). For the genotype frequencies, the ‘C’ allele was found in only a small proportion of the case population with the TC and CC genotypes representing only 11.4% and 5.7%, respectively. The TT genotype represented 100% of the control genotypes and 82.9% of the case genotypes (Figure 1). The ‘G’ allele of SNP rs8804236 was associated with diabetes in the Miniature Dachshund (Table 2). It represented 14% of the case alleles and 36% of the control alleles and the GG homozygous genotype was more common in the control population (29.6%) than in the cases (2.7%). The GA heterozygous genotype was more common in the cases (21.6%) than the controls (3.8%); the AA homozygous genotype represented 75.5% and 69.2% of the cases and controls respectively (Figure 1).

Three SNPs were associated with diabetes in the Cocker Spaniel breed (Table 2). For SNPs rs22302353 and rs22686871, the major allele homozygote (GG and TT respectively) was more common in the cases than the controls and for rs22686871, it represented 100% of the case genotypes, compared to 78.5% of the controls (Figure 1). For SNP rs24305581, the minor allele (‘A’) was found in 69.7% of the case genotypes (AA+AG) vs. 39.4% of the control genotypes and the GG genotype was more common in the controls (60.6%) than the cases (29.3%, Figure 1). The heterozygous genotype for this SNP represented more than 50% of the case genotypes but only 33.3% of the control genotypes. Analysis of genotype combinations across these three associated markers in this breed did not identify any genotype combinations that were more or less common in either the case or the control populations, indicating that the associations are independent of each other (data not shown).

## Discussion

Some cases of canine diabetes share several similarities with the monogenic forms of human diabetes, known as maturity onset diabetes of the young (MODY). To date, however, there is no reported evidence of monogenic diabetes association studies in dogs. We identified six canine allelic associations to genes that are causative for human monogenic forms of diabetes, but none of these associations can fully explain diabetes risk in any given dog breed. One gene (*ZFP57*) was associated with two different SNPs in two disparate breeds (Bichon Frise and Samoyed) and one breed (Cocker Spaniel) had an association with three SNPs from three different genes (*MTTL1, PAX4, INS*). The allele and genotype frequencies do not indicate that these associations explain the full susceptibility to canine breed-related diabetes and suggest that canine diabetes is a polygenic trait with multiple genes conferring susceptibility. They could suggest, however, that a proportion of the dogs within a breed have a monogenic form of diabetes and that the remainder of the breed have a polygenic form of the condition. This requires further investigation for better clarification.

Associations within the Cocker Spaniel breed could suggest that three types of monogenic diabetes co-exist within this breed if, for example, they were in LD with the causative SNP rather than being the actual causative SNP. This seems highly unlikely however, given the reported genetic uniformity of pedigree dog breeds, although genome-wide analysis (GWA) of Cocker Spaniels within our overall sample population has identified three distinct clusters of Cocker Spaniels when viewed on a multi-dimensional scaling plot (Jonathan Massey, unpublished data). The representation of three clusters following GWA indicates the presence of genetic stratification within the breed and could explain, in part, the association of three different genes, each representing a distinct type of diabetes within this breed in which the respective genes are contributory to diabetes susceptibility within a cluster but that other unidentified genes are also contributory. The lack of GWA data on the specific dogs used in this candidate gene study prevents the stratification of this cohort into genome-wide sub-populations but could be a useful investigation strategy for future studies.

Three of the associated SNPs were intronic (rs24305581, rs22302353, rs23892119, Table 2) and while the specific function of these SNPs is unknown at the current time, intronic SNPs are known to affect gene expression through regulatory elements and have been shown to activate cryptic splice sites, leading to alternative splicing [56].

The other three associated SNPs were synonymous coding SNPs (rs23901704, rs22686871 rs8804236, Table 2) and it is well documented that synonymous SNPs affect gene function through mRNA splicing and can also affect precursor mRNA splicing, RNA stability and structure and protein folding. These synonymous SNPs could, therefore, result in ectopic mRNA splice sites and generate null, antagonising or agonistic protein isoforms leading to the diabetic disease phenotype. They could equally affect mRNA stability and subsequently the amount of available protein transcript could be increased or decreased or they could result in defective protein that do not function properly.

The majority of breeds used in this analysis were not associated with the candidate genes that were selected. A monogenic type of diabetes could still exist within these breeds, but as yet the causative gene has not yet been identified. Alternatively it could be that the canine version of the causative gene was insufficiently annotated at the time of study and thus not included in the present study. An example of the latter is glucokinase which would be a good candidate gene, once the canine *GCK* sequence (XM_846042.2) and its chromosomal location have been better resolved (CanFam 3.1, http://www.ncbi.nlm.nih.gov/gene/606490).

The limited number of associations with monogenic diabetes susceptibility genes could also indicate that canine diabetes is largely polygenic in most breeds and could also be subject to environmental influences. The finding of a small number of significant gene associations, and even some of the associations not retained following statistical correction, may indicate that these genes represent small risk contributions to a larger undiscovered polygenetic aetiology. This is further supported by the allele frequencies of the non-associated breeds in the study which are provided as additional information (Additional file 1: Table S1) and show that the associated alleles are often present in the other breeds but do not reach significance. In some breeds, the allele that is associated with diabetes in one breed is found at equal frequency in cases and controls of a different breed. For example, SNP rs23901704 showed an association to diabetes in the Samoyed (Table 2) with the minor allele (C) being found at a frequency of 0.11 in the cases only. This same allele (C) was found at similar frequencies for both controls and cases respectively in the Border collie (0.2 and 0.17), Labrador (0.17 vs. 0.22), Miniature schnauzer (0.36 vs. 0.34), Samoyed (0.15 vs. 0.17) Springer Spaniel (0.08 vs. 0.12), West Highland white terrier (0.16 vs. 0.18) and Yorkshire terrier (0.09 vs. 0.06). Similar trends are seen for the other associated SNPs in the different breeds.

A number of immunity-related genes, including the dog leukocyte antigen (DLA) have already been associated with canine diabetes in some breeds [57–61]. This is not surprising, however, given the pathogenesis of the condition and the potential immune-mediated destruction of pancreatic beta cells. Many of the cytokine SNPs that have been associated with an increased risk of developing canine diabetes are from the Th2 subset [57–60]. This is important because the Th1-Th2 balance is considered to be instrumental in the development of this condition and diabetes is believed to be initiated by Th1 cytokines.

To achieve significant power in genome-wide association studies (GWAS) to identify genes and estimate the size of their contribution in complex polygenic conditions, human genetic analysis cohorts often require thousands of cases and controls. This is not the case in GWAS studies of pedigree dog breeds where the sample size may be as few as a hundred and power is increased when taking into account the small number of founder members for a given breed, the high level of inbreeding and the extended linkage disequilibrium and haplotype structure that exists in pedigree dog breeds [29]. It has been suggested that even fewer cases and an equal number of controls are sufficient to detect the disease allele for a simple Mendelian recessive trait [32]. With the exception of the Staffordshire bull terrier and Doberman, all of our breed cohorts contained more than 20 cases and controls, suggesting we should have had sufficient statistical power to detect associations for a monogenic disorder. The limited number of associations identified in this study is therefore most likely to be due to canine diabetes being a polygenic trait in most dog breeds.

This is the first multi-breed candidate gene analysis to investigate gene homologues in the dog that are equivalent to human monogenic forms of diabetes. We have evidence that some of the same genes that have been associated with susceptibility to MODY and NDM in humans are also associated with canine diabetes, although the number of breeds where this applies is limited and it is more likely that canine diabetes is a polygenic trait. Recruitment of cases or controls to such studies is difficult unless performed under a Home Office animal licence and most veterinary blood samples submitted to laboratories for DNA isolation are generally residual from other diagnostic tests.

## Conclusions

The underlying aetiology and pathogenesis of canine diabetes have not been fully established and while insulin deficiency is a consistent feature, it remains a heterogeneous condition. The presence of specific breed predispositions and phenotypes within breeds suggests an underlying genetic basis for diabetes susceptibility but this susceptibility varies between breeds and is likely to result from the interactions of multiple genes. The current study and previous candidate gene studies have identified breed-specific genetic associations with the condition, but none of the identified associations can fully explain canine diabetes susceptibility. Ongoing GWA studies are expected to identify new loci that will further explain the breed susceptibility to diabetes in dogs.

## Methods

### Study design

Blood samples from diabetic dogs were selected from the UK Canine Diabetes Register and Archive (Royal Veterinary College, University of London). Diagnosis of diabetes was based on consistent clinical signs (polyuria, polydipsia and weight loss) and documented hyperglycaemia (i.e. > 9 mmol/l) with glucosuria. Entire females were excluded from the study to eliminate dogs suffering from dioestrus diabetes, an insulin-resistance form of the disease. Dog samples representing seventeen breeds were selected from a larger collection of diabetic samples on the basis of providing a sample size n ≥ 15 in each breed group (Table 1). Breed-matched, control samples (without diabetes) were selected from a large archive of DNA samples ( http://www.liv.ac.uk/dna_archive_for_companion_animals/) UK DNA Archive for Companion Animals, Universities of Manchester and Liverpool) which collects samples from animals being treated for a range of conditions. For this cohort, controls were selected as samples from dogs that were older than 7 years of age and had been diagnosed with (and were responding to treatment for) non-autoimmune, non-endocrine conditions. Conditions permitted for inclusion were those where the clinical signs were least likely to present with a diabetic phenotype and included, but was not limited to, hip dysplasia, cruciate rupture and neurological dysfunction (epilepsy). It was not possible to test for hyperglycaemia in these retrospectively recruited samples. Control samples were selected from the same geographical region as that for the dogs with diabetes, wherever possible.

Sex and neutered status were not available for all of the control dogs thus eliminating the possibility of investigating sex bias and it was not possible to assess the relatedness of affected dogs as UK Kennel Club registration numbers were not available for the majority of dogs investigated. Allelic association was conducted in a breed-by-breed manner.

### DNA extraction

DNA was extracted from residual EDTA blood samples using either a standard phenol: chloroform method or a Qiagen QIAamp DNA Blood Midi Kit in accordance with the manufacturer’s instructions. DNA quality (A260:280) and concentration were measured using a NanoDrop ( http://www.nanodrop.com/). All samples had a 260:280 ratio between 1.6 and 1.9 were diluted to a final concentration of 5 ng/μl for SNP genotyping.

### Candidate gene and SNP selection

Nineteen candidate genes were chosen based on reported associations to human forms of monogenic diabetes (Table 3). A total of 65 SNPs were genotyped with SNP selection being prioritised for inclusion if they were non-synonymous or synonymous coding SNPs or were located in the 3’or 5’ UTR. Intronic SNPs were included in the absence of coding/UTR SNPs and were selected as those being closest to the intron/exon boundaries on the assumption that ‘within breed’ linkage disequilibrium would extend across the boundaries, as opposed to conservation or the presence of regulatory elements. The SNPs used in the analyses are shown in Table 3.

**Table 3:** **Genes and SNPs used in the study and type of human monogenic diabetes that has been associated with the gene**

Gene	Protein	SNP IDs		Type of monogenic diabetes in humans	Associated literature
ABCC8	Sulfonylurea receptor 1	rs9183439	rs22988565	PNDM, TNDM	[33]
		rs22993873	rs9044450		[34]
BLK	B lymphoid tyrosine kinase	rs23277058	rs23242723	MODY 11	[35]
		rs23228211	rs23268052		[36]
Cel	Carboxyl ester lipase	rs8843005	rs8843006	MODY 8 (with exocrine dysfunction)	[37]
		rs24549495			[38, 39]
EIF2AK3	Eukaryotic translation	rs22578314	rs9179252	PNDM (with epiphyseal dysplasia)	[40]
	initiation factor 2a kinase	rs22578182	rs22566811		
FOXP3	Forkhead box P3	rs24618205	rs24596299	PNDM (X-linked with immune-dysregulation, polyendocrinopathy, enteropathy)	[41]
		rs24612921			[42]
HNF1A	Hepatocyte nuclear factor 1a	rs23350532	rs9013694	MODY 3	[43]
		rs23309484			[20]
HNF1B	Hepatocyte nuclear factor 1b	rs24537168	rs24585301	MODY 5 (also with renal dysfunction, genital malformations) and PNDM	[44]
		rs24537175	rs24585484		
HNF4A	Hepatocyte nuclear factor 4a	rs23214782	rs23200327	MODY 1	[43]
		rs8804236	rs23200360		[45]
		rs23214781	rs9006559		[16]
INS	Insulin	rs22686871		PNDM, MODY	[23]
KLF11	Kruppel-like factor 11	rs22598321	rs8803647	MODY 7	[46]
MTTL1	Transfer RNA for protein	rs8648077	rs8884972	Mitochondrial diabetes (maternally transmitted with deafness)	[47]
	translation	rs24305581			[48]
PAX4	Paired box 4	rs22302371	rs22302353	MODY 9	[49]
PDX1		rs8837751	rs8837750	MODY 4, PNDM (with pancreatic agenesis)	[50]
	Insulin promoter factor 1		rs23247540		[21]
PTF1A	Pancreas-specific	rs8955054	rs8955053	PNDM (with pancreatic and cerebellar agenesis)	[51]
	transcription factor 1a	rs8955055			
RFX6	Regulatory factor X6	rs8928516	rs21958946	PNDM (with hypoplastic pancreas and gall bladder, intestinal atresia)	[52]
		rs21890992			
WFS1		rs24739532		PNDM (with diabetes insipidus, optic atrophy, deafness)	[53]
	Wolframin				[54]
ZFP57	Zinc finger protein 57	rs23901704	rs23892119	TNDM	[55]
		rs23892118	rs23901705		
ZAC1/PLAG1	Pleiomorphic adenoma gene-like 1	rs23483681		TNDM	

PNDM: Permanent neonatal diabetes; TNDM: Transient neonatal diabetes; MODY: Maturity onset diabetes of the young.

### Sequenom genotyping

Primers and probes were designed using Sequenom Assay Design software Version 3, and synthesised by Sigma-Aldrich (Poole, UK). Primers were diluted to 100 μM and plexes pooled to contain 500 nm of each forward and reverse primer. Probes were diluted to 400 μM and probe pools were split into four tiers dependent upon mass. Probe pools were split into four equal tiers containing 26 μl, 35 μl, 43 μl and 52 μl of probe (low to high mass), in a final volume of 1.5 ml.

PCR reactions contained 20 ng DNA plated into a 384 well plate. PCRs were performed in 5 μl volumes using an ABI 9700 cycler (384 well). Reactions contained 0.625 μl of 10× PCR buffer (with 20 mM MgCl_2_, Roche), 0.2 μl of MgCl_2_ (25 mM), 0.25μl of dNTPs (10 mM), 100 nM of forward and reverse primer plex, 0.1 μl FastStart Taq (5 U/μl, Roche) and were amplified as follows: 95°C for 5 minutes; 40 cycles of 95°C for 20 seconds, 56°C for 30 seconds, 72°C for 1 minute; 72°C for 3 minutes. Following PCR, reactions were treated with 0.3 U/μl shrimp alkaline phosphatase (SAP) to dephosphorylate remaining dNTPs. Reactions were incubated at 37°C for 40 minutes, and denatured at 85°C for 5 minutes. iPLEX primer extension was carried out using an ABI 9700 PCR engine. Reactions contained 0.22× iPLEX buffer, 1x iPLEX termination mix, primers adjusted for concentration using a four tier method (0.625 μM, 0.83 μM, 1.04 μM, and 1.25 μM) and 1× iPLEX enzyme, and were amplified as follows: 94°C for 30 seconds, 40 cycles of 94°C for 5 seconds, 5 cycles of 52°C for 5 seconds, 80°C for 5 seconds, and a final extension of 72°C for 3 minutes. Samples were diluted with 20 μl water and desalted using 6 mg resin before being centrifuged for 5 minutes at 4,000 rpm and spotted onto a SpectroCHIP using a Sequenom mass array nanodispenser (Samsung).

### Allelic association analyses

Association analyses and quality checks were carried out using PLINK [62]. Hardy-Weinberg equilibrium (HWE) was checked for each breed control group and call rates were determined for cases and controls for each breed, independently. SNPs in which the control population was out of HWE and/or the call rate was below 90% were excluded from the analyses. Samples were excluded if the call rate was below 90%. Permutation testing and Bonferroni correction were applied to raw p values and SNPs were deemed statistically significant if *p*_*corrected*_<0.05.

Markers showing allelic association were also tested for a genotype association.

## Electronic supplementary material

Additional file 1: Table S1: Minor allele frequencies for each breed. Table shows the minor allele, its frequency in controls and cases and the major allele for each marker in each breed. The number of controls and cases that were genotyped and passed QC is also shown. (DOCX 94 KB)
