# Supplementary material for: Searching for “monogenic diabetes” in dogs using a candidate gene approach
Source: Canine Genet Epidemiol. 2014 Jul 7;1:8. doi: 10.1186/2052-6687-1-8 (PMC4579387; doi:10.1186/2052-6687-1-8)
Supplement: Supplementary file 1 — Additional file 1: Table S1: Minor allele frequencies for each breed. Table shows the minor allele, its frequency in controls and cases and the major allele for each marker in each breed. The number of controls and cases that were genotyped and passed QC is also shown. (DOCX 94 KB) [file 40575_2014_8_MOESM1_ESM.docx]

Supplementary table: Minor allele frequencies for each breed. Table shows the minor allele, its frequency in controls and cases and the major allele for each marker in each breed. The number of controls and cases that were genotyped and passed QC is also shown.

|  | Bichon Frise | | | | Border collie | | | | Border terrier | | | | Cairn terrier | | | |
| --- | --- | --- | --- | --- | --- | --- | --- | --- | --- | --- | --- | --- | --- | --- | --- | --- |
|  | Minor allele | Frequency | | Major allele | Minor allele | Frequency | | Major allele | Minor allele | Frequency | | Major allele | Minor allele | Frequency | | Major allele |
| Marker |  | Controls n=29 | Cases  n=28 |  |  | Controls n=79 | Cases  n=76 |  |  | Controls n=25 | Cases  n=24 |  |  | Controls n=39 | Cases  n=42 |  |
| RS21890992 | G | 0.00 | 0.00 | A | G | 0.15 | 0.11 | A | G | 0.00 | 0.00 | A | G | 0.02 | 0.01 | A |
| RS21958946 | A | 0.00 | 0.00 | T | A | 0.17 | 0.12 | T | A | 0.00 | 0.00 | T | A | 0.26 | 0.23 | T |
| RS22302353 | A | 0.33 | 0.22 | G | A | 0.13 | 0.17 | G | A | 0.39 | 0.35 | G | A | 0.05 | 0.07 | G |
| RS22302371 | T | 0.09 | 0.09 | C | T | 0.01 | 0.04 | C | T | 0.02 | 0.02 | C | C | 0.43 | 0.36 | T |
| RS22566811 | A | 0.12 | 0.17 | G | A | 0.24 | 0.23 | G | G | 0.15 | 0.18 | A | G | 0.45 | 0.49 | A |
| RS22578182 | T | 0.00 | 0.00 | C | T | 0.02 | 0.03 | C | T | 0.00 | 0.02 | C | T | 0.05 | 0.07 | C |
| RS22578314 | G | 0.12 | 0.17 | A | G | 0.23 | 0.22 | A | A | 0.14 | 0.18 | G | A | 0.44 | 0.49 | G |
| RS22598321 | C | 0.00 | 0.00 | G | C | 0.00 | 0.00 | G | C | 0.00 | 0.00 | G | C | 0.00 | 0.00 | G |
| RS22686871 | *G | 0.38 | 0.48 | T | *G | 0.35 | 0.24 | T | G | 0.00 | 0.00 | T | T | 0.27 | 0.27 | G |
| RS22988565 | *G | 0.34 | 0.45 | A | G | 0.28 | 0.29 | A | A | 0.15 | 0.29 | G | G | 0.02 | 0.06 | A |
| RS22993873 | A | 0.00 | 0.00 | C | A | 0.00 | 0.00 | C | A | 0.00 | 0.00 | C | A | 0.00 | 0.00 | C |
| RS23200327 | C | 0.48 | 0.43 | G | G | 0.24 | 0.30 | C | C | 0.24 | 0.24 | G | G | 0.10 | 0.17 | C |
| RS23200360 | T | 0.17 | 0.18 | C | T | 0.27 | 0.24 | C | T | 0.16 | 0.06 | C | T | 0.08 | 0.12 | C |
| RS23214781 | G | 0.24 | 0.16 | A | G | 0.32 | 0.32 | A | G | 0.15 | 0.10 | A | G | 0.36 | 0.42 | A |
| RS23214782 | T | 0.40 | 0.31 | C | T | 0.39 | 0.33 | C | T | 0.15 | 0.10 | C | T | 0.41 | 0.42 | C |
| RS23228211 | C | 0.22 | 0.25 | T | C | 0.48 | 0.40 | T | T | 0.36 | 0.33 | C | T | 0.38 | 0.42 | C |
| RS23242723 | C | 0.12 | 0.17 | A | C | 0.34 | 0.32 | A | C | 0.50 | 0.34 | A | A | 0.36 | 0.43 | C |
| RS23247540 | T | 0.23 | 0.22 | C | *T | 0.40 | 0.35 | C | T | 0.23 | 0.23 | C | T | 0.24 | 0.23 | C |
| RS23268052 | *T | 0.50 | 0.48 | C | T | 0.39 | 0.42 | C | *T | 0.39 | 0.40 | C | T | 0.36 | 0.32 | C |
| RS23277058 | A | 0.00 | 0.00 | G | A | 0.00 | 0.00 | G | A | 0.00 | 0.00 | G | A | 0.00 | 0.00 | G |
| RS23309484 | G | 0.00 | 0.00 | C | G | 0.05 | 0.02 | C | G | 0.38 | 0.28 | C | C | 0.24 | 0.17 | G |
| RS23350532 | T | 0.36 | 0.41 | C | T | 0.10 | 0.08 | C | T | 0.31 | 0.22 | C | T | 0.12 | 0.07 | C |
| RS23483681 | T | 0.00 | 0.00 | C | T | 0.10 | 0.15 | C | T | 0.04 | 0.04 | C | T | 0.04 | 0.11 | C |
| RS23892118 | T | 0.02 | 0.00 | C | T | 0.01 | 0.00 | C | T | 0.12 | 0.09 | C | T | 0.03 | 0.01 | C |
| RS23892119 | C | 0.00 | 0.00 | T | C | 0.00 | 0.01 | T | C | 0.00 | 0.00 | T | C | 0.00 | 0.00 | T |
| RS23901704 | T | 0.24 | 0.00 | C | T | 0.20 | 0.17 | C | T | 0.10 | 0.00 | C | T | 0.03 | 0.00 | C |
| RS23901705 | A | 0.00 | 0.00 | G | A | 0.21 | 0.14 | G | A | 0.00 | 0.00 | G | A | 0.15 | 0.18 | G |
| RS24305581 | *A | 0.45 | 0.38 | G | A | 0.29 | 0.39 | G | A | 0.43 | 0.40 | G | G | 0.40 | 0.36 | A |
| RS24537168 | T | 0.22 | 0.05 | A | *T | 0.08 | 0.12 | A | A | 0.46 | 0.50 | T | T | 0.21 | 0.27 | A |
| RS24537175 | T | 0.36 | 0.40 | C | T | 0.44 | 0.47 | C | T | 0.22 | 0.24 | C | T | 0.41 | 0.36 | C |
| RS24549495 | A | 0.24 | 0.17 | G | A | 0.49 | 0.46 | G | A | 0.33 | 0.36 | G | A | 0.36 | 0.38 | G |
| RS24585301 | A | 0.31 | 0.36 | G | A | 0.01 | 0.03 | G | A | 0.00 | 0.04 | G | A | 0.02 | 0.01 | G |
| RS24585484 | T | 0.12 | 0.10 | C | T | 0.24 | 0.20 | C | T | 0.02 | 0.02 | C | T | 0.28 | 0.21 | C |
| RS24596299 | A | 0.00 | 0.00 | G | A | 0.00 | 0.00 | G | A | 0.00 | 0.00 | G | A | 0.00 | 0.00 | G |
| RS24612921 | A | 0.00 | 0.00 | C | A | 0.00 | 0.00 | C | A | 0.00 | 0.00 | C | A | 0.00 | 0.00 | C |
| RS24618205 | G | 0.00 | 0.00 | T | G | 0.00 | 0.00 | T | G | 0.00 | 0.00 | T | G | 0.00 | 0.00 | T |
| RS24739532 | A | 0.00 | 0.00 | C | A | 0.00 | 0.00 | C | A | 0.00 | 0.00 | C | A | 0.00 | 0.00 | C |
| RS8648077 | T | 0.00 | 0.00 | C | T | 0.02 | 0.01 | C | T | 0.00 | 0.00 | C | T | 0.02 | 0.00 | C |
| RS8803647 | I | 0.00 | 0.00 | D | I | 0.00 | 0.00 | D | I | 0.00 | 0.00 | D | I | 0.00 | 0.00 | D |
| RS8804236 | G | 0.19 | 0.33 | A | G | 0.35 | 0.34 | A | A | 0.15 | 0.12 | G | A | 0.38 | 0.49 | G |
| RS8837750 | A | 0.02 | 0.02 | G | A | 0.01 | 0.01 | G | A | 0.20 | 0.13 | G | A | 0.04 | 0.01 | G |
| RS8837751 | T | 0.30 | 0.21 | C | T | 0.04 | 0.03 | C | T | 0.48 | 0.48 | C | T | 0.29 | 0.26 | C |
| RS8843005 | T | 0.22 | 0.32 | C | T | 0.01 | 0.01 | C | T | 0.00 | 0.00 | C | T | 0.00 | 0.00 | C |
| RS8843006 | T | 0.29 | 0.24 | C | C | 0.23 | 0.31 | T | T | 0.37 | 0.38 | C | C | 0.49 | 0.49 | T |
| RS8884972 | *A | 0.50 | 0.46 | C | C | 0.45 | 0.46 | A | A | 0.37 | 0.37 | C | A | 0.14 | 0.08 | C |
| RS8928516 | I | 0.00 | 0.00 | D | I | 0.00 | 0.00 | D | I | 0.00 | 0.00 | D | I | 0.02 | 0.00 | D |
| RS8955053 | I | 0.16 | 0.09 | D | I | 0.36 | 0.38 | D | I | 0.46 | 0.38 | D | I | 0.15 | 0.18 | D |
| RS8955054 | *A | 0.08 | 0.04 | T | *A | 0.17 | 0.18 | T | *A | 0.40 | 0.19 | T | *A | 0.10 | 0.09 | T |
| RS8955055 | I | 0.17 | 0.09 | D | I | 0.37 | 0.39 | D | I | 0.46 | 0.38 | D | I | 0.22 | 0.28 | D |
| RS9006559 | D | 0.00 | 0.00 | I | D | 0.00 | 0.01 | I | D | 0.00 | 0.00 | I | D | 0.00 | 0.00 | I |
| RS9013694 | A | 0.02 | 0.00 | G | A | 0.12 | 0.17 | G | *A | 0.33 | 0.20 | G | A | 0.09 | 0.08 | G |
| RS9044450 | T | 0.02 | 0.00 | C | T | 0.01 | 0.00 | C | T | 0.00 | 0.00 | C | T | 0.01 | 0.00 | C |
| RS9183439 | A | 0.00 | 0.00 | C | A | 0.00 | 0.00 | C | A | 0.00 | 0.00 | C | A | 0.00 | 0.00 | C |

Supplementary table continued

|  | Cavalier King Charles spaniel | | | | Cocker spaniel | | | | Miniature Dachshund | | | | Doberman | | | |
| --- | --- | --- | --- | --- | --- | --- | --- | --- | --- | --- | --- | --- | --- | --- | --- | --- |
|  | Minor allele | Frequency | | Major allele | Minor allele | Frequency | | Major allele | Minor allele | Frequency | | Major allele | Minor allele | Frequency | | Major allele |
| Marker |  | Controls n=46 | Cases  n=50 |  |  | Controls n=66 | Cases  n=58 |  |  | Controls n=39 | Cases  n=37 |  |  | Controls n=18 | Cases  n=17 |  |
| RS21890992 | G | 0.24 | 0.25 | A | G | 0.27 | 0.40 | A | *G | 0.19 | 0.23 | A | G | 0.00 | 0.00 | A |
| RS21958946 | A | 0.24 | 0.25 | T | A | 0.30 | 0.40 | T | A | 0.31 | 0.31 | T | A | 0.00 | 0.00 | T |
| RS22302353 | G | 0.42 | 0.28 | A | A | 0.33 | 0.09 | G | A | 0.50 | 0.41 | G | A | 0.29 | 0.31 | G |
| RS22302371 | T | 0.13 | 0.13 | C | T | 0.08 | 0.07 | C | T | 0.08 | 0.04 | C | T | 0.45 | 0.32 | C |
| RS22566811 | G | 0.47 | 0.35 | A | *G | 0.46 | 0.39 | A | *G | 0.29 | 0.39 | A | A | 0.11 | 0.19 | G |
| RS22578182 | T | 0.21 | 0.28 | C | T | 0.00 | 0.00 | C | *T | 0.04 | 0.03 | C | T | 0.05 | 0.00 | C |
| RS22578314 | G | 0.35 | 0.35 | A | *G | 0.45 | 0.39 | A | G | 0.26 | 0.38 | A | G | 0.08 | 0.19 | A |
| RS22598321 | C | 0.00 | 0.00 | G | C | 0.00 | 0.00 | G | C | 0.00 | 0.00 | G | C | 0.00 | 0.00 | G |
| RS22686871 | T | 0.44 | 0.34 | G | G | 0.13 | 0.00 | T | G | 0.05 | 0.15 | T | G | 0.11 | 0.09 | T |
| RS22988565 | A | 0.31 | 0.29 | G | G | 0.33 | 0.27 | A | *G | 0.31 | 0.50 | A | A | 0.13 | 0.06 | G |
| RS22993873 | A | 0.00 | 0.00 | C | A | 0.00 | 0.00 | C | A | 0.00 | 0.00 | C | A | 0.00 | 0.00 | C |
| RS23200327 | C | 0.21 | 0.16 | G | G | 0.14 | 0.08 | C | G | 0.24 | 0.08 | C | G | 0.47 | 0.38 | C |
| RS23200360 | T | 0.15 | 0.13 | C | T | 0.15 | 0.04 | C | C | 0.36 | 0.21 | T | T | 0.00 | 0.00 | C |
| RS23214781 | G | 0.05 | 0.06 | A | G | 0.11 | 0.03 | A | A | 0.42 | 0.22 | G | G | 0.08 | 0.14 | A |
| RS23214782 | T | 0.05 | 0.06 | C | *T | 0.16 | 0.04 | C | C | 0.35 | 0.18 | T | T | 0.08 | 0.14 | C |
| RS23228211 | C | 0.05 | 0.05 | T | *C | 0.44 | 0.35 | T | C | 0.37 | 0.42 | T | C | 0.00 | 0.03 | T |
| RS23242723 | C | 0.03 | 0.00 | A | C | 0.27 | 0.30 | A | C | 0.08 | 0.12 | A | C | 0.00 | 0.00 | A |
| RS23247540 | *T | 0.44 | 0.48 | C | *T | 0.43 | 0.37 | C | *T | 0.37 | 0.37 | C | T | 0.06 | 0.03 | C |
| RS23268052 | *T | 0.48 | 0.50 | C | *T | 0.43 | 0.41 | C | *T | 0.50 | 0.46 | C | *T | 0.50 | 0.50 | C |
| RS23277058 | A | 0.00 | 0.00 | G | A | 0.00 | 0.00 | G | A | 0.00 | 0.00 | G | A | 0.00 | 0.00 | G |
| RS23309484 | G | 0.13 | 0.15 | C | G | 0.05 | 0.01 | C | G | 0.08 | 0.14 | C | G | 0.03 | 0.03 | C |
| RS23350532 | T | 0.25 | 0.19 | C | T | 0.31 | 0.22 | C | T | 0.30 | 0.12 | C | T | 0.34 | 0.17 | C |
| RS23483681 | T | 0.06 | 0.14 | C | T | 0.25 | 0.25 | C | T | 0.18 | 0.10 | C | T | 0.00 | 0.00 | C |
| RS23892118 | T | 0.10 | 0.09 | C | T | 0.09 | 0.05 | C | T | 0.01 | 0.00 | C | T | 0.00 | 0.00 | C |
| RS23892119 | C | 0.00 | 0.00 | T | C | 0.01 | 0.00 | T | C | 0.08 | 0.07 | T | C | 0.00 | 0.00 | T |
| RS23901704 | T | 0.00 | 0.01 | C | T | 0.02 | 0.00 | C | T | 0.29 | 0.18 | C | T | 0.05 | 0.00 | C |
| RS23901705 | A | 0.00 | 0.00 | G | A | 0.12 | 0.08 | G | A | 0.17 | 0.21 | G | A | 0.03 | 0.03 | G |
| RS24305581 | A | 0.03 | 0.03 | G | A | 0.23 | 0.44 | G | A | 0.49 | 0.49 | G | G | 0.13 | 0.28 | A |
| RS24537168 | T | 0.02 | 0.01 | A | *T | 0.25 | 0.08 | A | T | 0.15 | 0.10 | A | T | 0.08 | 0.06 | A |
| RS24537175 | C | 0.42 | 0.40 | T | T | 0.27 | 0.45 | C | C | 0.27 | 0.24 | T | C | 0.45 | 0.19 | T |
| RS24549495 | A | 0.06 | 0.07 | G | A | 0.13 | 0.08 | G | A | 0.33 | 0.45 | G | A | 0.19 | 0.19 | G |
| RS24585301 | A | 0.26 | 0.26 | G | *A | 0.23 | 0.13 | G | A | 0.01 | 0.00 | G | A | 0.16 | 0.08 | G |
| RS24585484 | C | 0.50 | 0.47 | T | T | 0.28 | 0.28 | C | T | 0.08 | 0.13 | C | T | 0.03 | 0.06 | C |
| RS24596299 | A | 0.00 | 0.00 | G | A | 0.00 | 0.00 | G | A | 0.00 | 0.00 | G | A | 0.00 | 0.00 | G |
| RS24612921 | A | 0.00 | 0.00 | C | A | 0.00 | 0.00 | C | A | 0.00 | 0.00 | C | A | 0.00 | 0.00 | C |
| RS24618205 | G | 0.00 | 0.00 | T | G | 0.00 | 0.00 | T | G | 0.00 | 0.00 | T | G | 0.00 | 0.00 | T |
| RS24739532 | A | 0.00 | 0.00 | C | A | 0.00 | 0.00 | C | A | 0.00 | 0.00 | C | A | 0.00 | 0.00 | C |
| RS8648077 | T | 0.00 | 0.00 | C | T | 0.00 | 0.00 | C | T | 0.00 | 0.00 | C | T | 0.00 | 0.00 | C |
| RS8803647 | I | 0.00 | 0.00 | D | I | 0.00 | 0.00 | D | I | 0.00 | 0.00 | D | I | 0.00 | 0.00 | D |
| RS8804236 | A | 0.23 | 0.16 | G | G | 0.17 | 0.06 | A | G | 0.36 | 0.14 | A | G | 0.47 | 0.39 | A |
| RS8837750 | A | 0.00 | 0.00 | G | A | 0.00 | 0.00 | G | A | 0.00 | 0.00 | G | A | 0.00 | 0.00 | G |
| RS8837751 | T | 0.01 | 0.00 | C | T | 0.05 | 0.02 | C | T | 0.03 | 0.01 | C | T | 0.00 | 0.00 | C |
| RS8843005 | T | 0.00 | 0.00 | C | T | 0.00 | 0.00 | C | T | 0.00 | 0.00 | C | T | 0.00 | 0.00 | C |
| RS8843006 | T | 0.07 | 0.07 | C | T | 0.23 | 0.23 | C | T | 0.32 | 0.49 | C | T | 0.26 | 0.26 | C |
| RS8884972 | A | 0.00 | 0.00 | C | A | 0.42 | 0.29 | C | A | 0.16 | 0.09 | C | A | 0.08 | 0.22 | C |
| RS8928516 | I | 0.00 | 0.00 | D | I | 0.00 | 0.02 | D | I | 0.00 | 0.00 | D | I | 0.00 | 0.00 | D |
| RS8955053 | I | 0.00 | 0.05 | D | I | 0.17 | 0.30 | D | I | 0.01 | 0.00 | D | D | 0.37 | 0.33 | I |
| RS8955054 | A | 0.00 | 0.00 | T | A | 0.05 | 0.11 | T | A | 0.00 | 0.00 | T | *A | 0.28 | 0.50 | T |
| RS8955055 | I | 0.00 | 0.05 | D | I | 0.20 | 0.30 | D | I | 0.01 | 0.00 | D | I | 0.11 | 0.06 | D |
| RS9006559 | D | 0.01 | 0.00 | I | D | 0.00 | 0.02 | I | D | 0.00 | 0.00 | I | D | 0.00 | 0.00 | I |
| RS9013694 | A | 0.00 | 0.00 | G | *A | 0.12 | 0.03 | G | A | 0.22 | 0.19 | G | A | 0.13 | 0.06 | G |
| RS9044450 | T | 0.00 | 0.00 | C | T | 0.00 | 0.00 | C | T | 0.00 | 0.01 | C | T | 0.00 | 0.00 | C |
| RS9183439 | A | 0.00 | 0.00 | C | A | 0.00 | 0.00 | C | A | 0.00 | 0.00 | C | A | 0.00 | 0.00 | C |

Supplementary table continued

|  | Jack Russell terrier | | | | Labrador | | | | Miniature Schnauzer | | | | Samoyed | | | |
| --- | --- | --- | --- | --- | --- | --- | --- | --- | --- | --- | --- | --- | --- | --- | --- | --- |
|  | Minor allele | Frequency | | Major allele | Minor allele | Frequency | | Major allele | Minor allele | Frequency | | Major allele | Minor allele | Frequency | | Major allele |
| Marker |  | Controls n=54 | Cases  n=58 |  |  | Controls n=155 | Cases n=136 |  |  | Controls n=29 | Cases  n=29 |  |  | Controls n=74 | Cases  n=40 |  |
| RS21890992 | G | 0.09 | 0.08 | A | G | 0.11 | 0.11 | A | G | 0.00 | 0.00 | A | G | 0.04 | 0.07 | A |
| RS21958946 | A | 0.11 | 0.10 | T | A | 0.11 | 0.11 | T | A | 0.00 | 0.00 | T | A | 0.12 | 0.08 | T |
| RS22302353 | A | 0.46 | 0.46 | G | A | 0.45 | 0.42 | G | A | 0.17 | 0.08 | G | A | 0.07 | 0.16 | G |
| RS22302371 | T | 0.18 | 0.14 | C | T | 0.00 | 0.01 | C | C | 0.20 | 0.09 | T | T | 0.13 | 0.16 | C |
| RS22566811 | G | 0.46 | 0.43 | A | G | 0.29 | 0.32 | A | G | 0.50 | 0.35 | A | G | 0.20 | 0.20 | A |
| RS22578182 | T | 0.02 | 0.10 | C | T | 0.10 | 0.12 | C | T | 0.00 | 0.00 | C | *T | 0.14 | 0.18 | C |
| RS22578314 | G | 0.44 | 0.49 | A | A | 0.39 | 0.44 | G | A | 0.48 | 0.34 | G | A | 0.35 | 0.38 | G |
| RS22598321 | C | 0.00 | 0.00 | G | C | 0.00 | 0.00 | G | C | 0.00 | 0.00 | G | C | 0.00 | 0.00 | G |
| RS22686871 | *G | 0.24 | 0.24 | T | G | 0.19 | 0.16 | T | G | 0.02 | 0.00 | T | G | 0.40 | 0.29 | T |
| RS22988565 | G | 0.48 | 0.34 | A | G | 0.25 | 0.24 | A | A | 0.35 | 0.28 | G | A | 0.32 | 0.27 | G |
| RS22993873 | A | 0.00 | 0.00 | C | A | 0.00 | 0.00 | C | A | 0.00 | 0.00 | C | A | 0.00 | 0.00 | C |
| RS23200327 | G | 0.18 | 0.28 | C | G | 0.39 | 0.42 | C | G | 0.03 | 0.06 | C | G | 0.00 | 0.00 | C |
| RS23200360 | *T | 0.38 | 0.33 | C | T | 0.07 | 0.07 | C | T | 0.02 | 0.07 | C | T | 0.08 | 0.05 | C |
| RS23214781 | *G | 0.33 | 0.25 | A | G | 0.03 | 0.04 | A | G | 0.18 | 0.10 | A | A | 0.38 | 0.30 | G |
| RS23214782 | *T | 0.34 | 0.27 | C | T | 0.03 | 0.05 | C | T | 0.18 | 0.16 | C | C | 0.35 | 0.29 | T |
| RS23228211 | C | 0.30 | 0.31 | T | C | 0.12 | 0.18 | T | T | 0.45 | 0.40 | C | C | 0.23 | 0.27 | T |
| RS23242723 | C | 0.18 | 0.16 | A | C | 0.01 | 0.03 | A | C | 0.20 | 0.23 | A | C | 0.17 | 0.12 | A |
| RS23247540 | T | 0.20 | 0.31 | C | *T | 0.28 | 0.31 | C | T | 0.02 | 0.02 | C | T | 0.07 | 0.20 | C |
| RS23268052 | *T | 0.46 | 0.47 | C | *T | 0.50 | 0.49 | C | *T | 0.38 | 0.37 | C | T | 0.49 | 0.44 | C |
| RS23277058 | A | 0.00 | 0.00 | G | A | 0.00 | 0.00 | G | A | 0.00 | 0.00 | G | A | 0.00 | 0.00 | G |
| RS23309484 | G | 0.10 | 0.15 | C | G | 0.26 | 0.24 | C | G | 0.05 | 0.00 | C | G | 0.14 | 0.16 | C |
| RS23350532 | *T | 0.28 | 0.40 | C | C | 0.28 | 0.34 | T | T | 0.07 | 0.10 | C | T | 0.29 | 0.32 | C |
| RS23483681 | T | 0.05 | 0.08 | C | T | 0.02 | 0.02 | C | T | 0.00 | 0.00 | C | T | 0.01 | 0.00 | C |
| RS23892118 | T | 0.02 | 0.03 | C | T | 0.05 | 0.06 | C | T | 0.02 | 0.00 | C | T | 0.01 | 0.05 | C |
| RS23892119 | *C | 0.07 | 0.02 | T | *C | 0.03 | 0.04 | T | C | 0.00 | 0.02 | T | C | 0.00 | 0.11 | T |
| RS23901704 | T | 0.23 | 0.10 | C | T | 0.17 | 0.22 | C | T | 0.36 | 0.34 | C | T | 0.15 | 0.17 | C |
| RS23901705 | A | 0.13 | 0.16 | G | A | 0.01 | 0.00 | G | A | 0.37 | 0.40 | G | A | 0.20 | 0.23 | G |
| RS24305581 | A | 0.31 | 0.19 | G | A | 0.15 | 0.20 | G | *A | 0.07 | 0.00 | G | G | 0.17 | 0.23 | A |
| RS24537168 | T | 0.14 | 0.18 | A | T | 0.14 | 0.08 | A | T | 0.43 | 0.27 | A | T | 0.00 | 0.00 | A |
| RS24537175 | T | 0.47 | 0.48 | C | C | 0.43 | 0.36 | T | C | 0.47 | 0.26 | T | T | 0.43 | 0.49 | C |
| RS24549495 | G | 0.47 | 0.45 | A | A | 0.24 | 0.25 | G | A | 0.17 | 0.25 | G | A | 0.36 | 0.26 | G |
| RS24585301 | A | 0.12 | 0.12 | G | A | 0.00 | 0.00 | G | A | 0.00 | 0.00 | G | A | 0.00 | 0.00 | G |
| RS24585484 | T | 0.15 | 0.20 | C | T | 0.06 | 0.03 | C | T | 0.05 | 0.13 | C | T | 0.43 | 0.27 | C |
| RS24596299 | A | 0.00 | 0.00 | G | A | 0.00 | 0.00 | G | A | 0.00 | 0.00 | G | *A | 0.00 | 0.00 | G |
| RS24612921 | A | 0.00 | 0.00 | C | A | 0.00 | 0.00 | C | A | 0.00 | 0.00 | C | A | 0.00 | 0.00 | C |
| RS24618205 | G | 0.00 | 0.00 | T | G | 0.00 | 0.00 | T | G | 0.00 | 0.00 | T | G | 0.00 | 0.00 | T |
| RS24739532 | A | 0.00 | 0.00 | C | A | 0.00 | 0.00 | C | A | 0.00 | 0.00 | C | A | 0.00 | 0.00 | C |
| RS8648077 | T | 0.00 | 0.00 | C | T | 0.01 | 0.01 | C | T | 0.00 | 0.00 | C | T | 0.01 | 0.00 | C |
| RS8803647 | I | 0.00 | 0.00 | D | I | 0.00 | 0.00 | D | I | 0.00 | 0.00 | D | I | 0.00 | 0.00 | D |
| RS8804236 | G | 0.24 | 0.35 | A | A | 0.32 | 0.25 | G | G | 0.07 | 0.13 | A | G | 0.29 | 0.26 | A |
| RS8837750 | A | 0.00 | 0.02 | G | A | 0.11 | 0.06 | G | A | 0.00 | 0.00 | G | A | 0.01 | 0.00 | G |
| RS8837751 | T | 0.19 | 0.21 | C | T | 0.31 | 0.29 | C | T | 0.00 | 0.00 | C | T | 0.09 | 0.07 | C |
| RS8843005 | T | 0.00 | 0.00 | C | *T | 0.01 | 0.00 | C | T | 0.00 | 0.00 | C | T | 0.00 | 0.00 | C |
| RS8843006 | C | 0.48 | 0.40 | T | T | 0.26 | 0.26 | C | T | 0.29 | 0.32 | C | T | 0.48 | 0.37 | C |
| RS8884972 | *A | 0.09 | 0.18 | C | A | 0.42 | 0.41 | C | A | 0.45 | 0.47 | C | A | 0.05 | 0.10 | C |
| RS8928516 | I | 0.00 | 0.00 | D | I | 0.00 | 0.01 | D | I | 0.00 | 0.00 | D | I | 0.01 | 0.00 | D |
| RS8955053 | I | 0.13 | 0.13 | D | *I | 0.13 | 0.09 | D | I | 0.25 | 0.25 | D | D | 0.44 | 0.33 | I |
| RS8955054 | A | 0.00 | O.07 | T | *A | 0.04 | 0.01 | T | *A | 0.19 | 0.15 | T | *A | 0.42 | 0.45 | T |
| RS8955055 | I | 0.13 | 0.14 | D | *I | 0.13 | 0.07 | D | *I | 0.34 | 0.40 | D | I | 0.04 | 0.08 | D |
| RS9006559 | D | 0.00 | 0.01 | I | D | 0.00 | 0.01 | I | D | 0.00 | 0.00 | I | D | 0.00 | 0.00 | I |
| RS9013694 | A | 0.06 | 0.06 | G | A | 0.05 | 0.04 | G | A | 0.00 | 0.00 | G | A | 0.15 | 0.32 | G |
| RS9044450 | T | 0.00 | 0.00 | C | T | 0.00 | 0.00 | C | T | 0.00 | 0.00 | C | T | 0.03 | 0.03 | C |
| RS9183439 | A | 0.00 | 0.00 | C | A | 0.00 | 0.00 | C | A | 0.00 | 0.00 | C | A | 0.00 | 0.00 | C |

Supplementary table continued

|  | Staffordshire bull terrier | | | | Springer spaniel | | | | Tibetan terrier | | | | West Highland white terrier | | | | Yorkshire terrier | | | |
| --- | --- | --- | --- | --- | --- | --- | --- | --- | --- | --- | --- | --- | --- | --- | --- | --- | --- | --- | --- | --- |
|  | Minor allele | Frequency | | Major allele | Minor allele | Frequency | | Major allele | Minor allele | Frequency | | Major allele | Minor allele | Frequency | | Major allele | Minor allele | Frequency | | Major allele |
| Marker |  | Controls n=15 | Cases  n=14 |  |  | Controls n=25 | Cases  n=21 |  |  | Controls n=30 | Cases  n=30 |  |  | Controls n=129 | Cases n=123 |  |  | Controls n=75 | Cases  n=76 |  |
| RS21890992 | G | 0.00 | 0.04 | A | *G | 0.22 | 0.20 | A | G | 0.05 | 0.00 | A | G | 0.00 | 0.00 | A | *G | 0.06 | 0.06 | A |
| RS21958946 | A | 0.00 | 0.04 | T | A | 0.34 | 0.34 | T | A | 0.37 | 0.40 | T | A | 0.45 | 0.44 | T | *A | 0.13 | 0.16 | T |
| RS22302353 | G | 0.33 | 0.50 | A | *G | 0.32 | 0.36 | A | G | 0.30 | 0.23 | A | A | 0.07 | 0.06 | G | G | 0.39 | 0.40 | A |
| RS22302371 | T | 0.00 | 0.04 | C | T | 0.00 | 0.02 | C | T | 0.07 | 0.12 | C | C | 0.17 | 0.14 | T | T | 0.06 | 0.06 | C |
| RS22566811 | A | 0.43 | 0.43 | G | A | 0.14 | 0.24 | G | A | 0.43 | 0.37 | G | A | 0.23 | 0.22 | G | A | 0.41 | 0.45 | G |
| RS22578182 | T | 0.30 | 0.14 | C | T | 0.02 | 0.11 | C | T | 0.15 | 0.07 | C | *T | 0.18 | 0.13 | C | T | 0.01 | 0.04 | C |
| RS22578314 | G | 0.13 | 0.29 | A | G | 0.12 | 0.14 | A | G | 0.33 | 0.35 | A | G | 0.20 | 0.20 | A | G | 0.38 | 0.40 | A |
| RS22598321 | C | 0.00 | 0.00 | G | C | 0.00 | 0.00 | G | C | 0.00 | 0.00 | G | C | 0.00 | 0.00 | G | C | 0.00 | 0.00 | G |
| RS22686871 | G | 0.43 | 0.18 | T | G | 0.12 | 0.07 | T | *G | 0.35 | 0.41 | T | T | 0.35 | 0.39 | G | *G | 0.15 | 0.07 | T |
| RS22988565 | A | 0.40 | 0.50 | G | G | 0.48 | 0.30 | A | A | 0.48 | 0.48 | G | G | 0.19 | 0.19 | A | A | 0.42 | 0.37 | G |
| RS22993873 | A | 0.00 | 0.00 | C | A | 0.00 | 0.00 | C | A | 0.00 | 0.00 | C | A | 0.00 | 0.00 | C | A | 0.00 | 0.00 | C |
| RS23200327 | G | 0.10 | 0.07 | C | G | 0.42 | 0.38 | C | G | 0.07 | 0.00 | C | G | 0.00 | 0.01 | C | G | 0.03 | 0.05 | C |
| RS23200360 | T | 0.42 | 0.46 | C | T | 0.33 | 0.30 | C | T | 0.43 | 0.34 | C | T | 0.04 | 0.07 | C | T | 0.21 | 0.22 | C |
| RS23214781 | G | 0.43 | 0.46 | A | G | 0.12 | 0.26 | A | A | 0.50 | 0.25 | G | G | 0.33 | 0.35 | A | G | 0.49 | 0.43 | A |
| RS23214782 | T | 0.43 | 0.46 | C | T | 0.38 | 0.48 | C | C | 0.47 | 0.22 | T | T | 0.36 | 0.39 | C | T | 0.49 | 0.43 | C |
| RS23228211 | C | 0.47 | 0.36 | T | C | 0.04 | 0.02 | T | T | 0.18 | 0.08 | C | *C | 0.08 | 0.05 | T | C | 0.26 | 0.34 | T |
| RS23242723 | C | 0.17 | 0.07 | A | C | 0.04 | 0.00 | A | A | 0.20 | 0.07 | C | C | 0.07 | 0.03 | A | C | 0.11 | 0.13 | A |
| RS23247540 | T | 0.18 | 0.35 | C | T | 0.02 | 0.02 | C | T | 0.02 | 0.03 | C | *T | 0.39 | 0.40 | C | *T | 0.32 | 0.34 | C |
| RS23268052 | *T | 0.46 | 0.46 | C | *T | 0.50 | 0.50 | C | T | 0.33 | 0.28 | C | *T | 0.50 | 0.48 | C | *T | 0.48 | 0.49 | C |
| RS23277058 | A | 0.00 | 0.00 | G | A | 0.00 | 0.00 | G | A | 0.00 | 0.00 | G | A | 0.00 | 0.00 | G | A | 0.00 | 0.00 | G |
| RS23309484 | G | 0.47 | 0.32 | C | G | 0.25 | 0.12 | C | G | 0.00 | 0.05 | C | C | 0.30 | 0.27 | G | G | 0.41 | 0.45 | C |
| RS23350532 | C | 0.23 | 0.29 | T | *T | 0.26 | 0.31 | C | T | 0.17 | 0.20 | C | *T | 0.23 | 0.19 | C | T | 0.43 | 0.36 | C |
| RS23483681 | T | 0.00 | 0.07 | C | T | 0.32 | 0.19 | C | T | 0.00 | 0.00 | C | T | 0.00 | 0.00 | C | T | 0.01 | 0.03 | C |
| RS23892118 | T | 0.00 | 0.00 | C | T | 0.02 | 0.03 | C | T | 0.00 | 0.00 | C | T | 0.16 | 0.13 | C | *T | 0.01 | 0.02 | C |
| RS23892119 | C | 0.00 | 0.00 | T | C | 0.00 | 0.03 | T | C | 0.00 | 0.00 | T | *C | 0.09 | 0.09 | T | C | 0.06 | 0.10 | T |
| RS23901704 | T | 0.10 | 0.04 | C | T | 0.08 | 0.12 | C | T | 0.10 | 0.06 | C | T | 0.16 | 0.18 | C | T | 0.09 | 0.06 | C |
| RS23901705 | A | 0.00 | 0.00 | G | A | 0.04 | 0.05 | G | A | 0.15 | 0.17 | G | A | 0.03 | 0.01 | G | A | 0.02 | 0.01 | G |
| RS24305581 | A | 0.17 | 0.29 | G | A | 0.20 | 0.11 | G | G | 0.42 | 0.43 | A | G | 0.23 | 0.30 | A | G | 0.49 | 0.44 | A |
| RS24537168 | T | 0.17 | 0.21 | A | T | 0.24 | 0.11 | A | T | 0.38 | 0.48 | A | T | 0.00 | 0.01 | A | T | 0.03 | 0.05 | A |
| RS24537175 | T | 0.30 | 0.11 | C | T | 0.20 | 0.11 | C | T | 0.23 | 0.27 | C | T | 0.27 | 0.19 | C | T | 0.36 | 0.42 | C |
| RS24549495 | A | 0.47 | 0.39 | G | A | 0.32 | 0.32 | G | G | 0.25 | 0.07 | A | A | 0.30 | 0.26 | G | A | 0.30 | 0.36 | G |
| RS24585301 | A | 0.07 | 0.00 | G | A | 0.04 | 0.09 | G | G | 0.40 | 0.32 | A | A | 0.00 | 0.01 | G | A | 0.45 | 0.44 | G |
| RS24585484 | T | 0.03 | 0.07 | C | C | 0.46 | 0.48 | T | T | 0.10 | 0.03 | C | T | 0.06 | 0.02 | C | T | 0.11 | 0.13 | C |
| RS24596299 | A | 0.00 | 0.00 | G | A | 0.00 | 0.00 | G | A | 0.00 | 0.00 | G | A | 0.00 | 0.00 | G | A | 0.00 | 0.00 | G |
| RS24612921 | A | 0.00 | 0.00 | C | A | 0.00 | 0.00 | C | A | 0.00 | 0.00 | C | A | 0.00 | 0.00 | C | A | 0.00 | 0.00 | C |
| RS24618205 | G | 0.00 | 0.00 | T | G | 0.00 | 0.00 | T | *G | 0.00 | 0.00 | T | G | 0.00 | 0.00 | T | G | 0.00 | 0.00 | T |
| RS24739532 | A | 0.00 | 0.00 | C | A | 0.00 | 0.00 | C | A | 0.00 | 0.00 | C | A | 0.00 | 0.00 | C | A | 0.00 | 0.00 | C |
| RS8648077 | T | 0.00 | 0.00 | C | T | 0.04 | 0.00 | C | T | 0.00 | 0.00 | C | T | 0.00 | 0.00 | C | T | 0.00 | 0.00 | C |
| RS8803647 | I | 0.00 | 0.00 | D | I | 0.00 | 0.00 | D | I | 0.00 | 0.00 | D | I | 0.00 | 0.00 | D | I | 0.00 | 0.00 | D |
| RS8804236 | G | 0.20 | 0.18 | A | *G | 0.36 | 0.39 | A | G | 0.12 | 0.05 | A | G | 0.42 | 0.40 | A | G | 0.03 | 0.04 | A |
| RS8837750 | A | 0.00 | 0.00 | G | A | 0.07 | 0.00 | G | A | 0.00 | 0.02 | G | A | 0.04 | 0.02 | G | A | 0.01 | 0.01 | G |
| RS8837751 | T | 0.00 | 0.04 | C | T | 0.24 | 0.33 | C | T | 0.07 | 0.12 | C | T | 0.20 | 0.22 | C | T | 0.03 | 0.04 | C |
| RS8843005 | T | 0.00 | 0.00 | C | T | 0.00 | 0.00 | C | T | 0.00 | 0.00 | C | T | 0.00 | 0.00 | C | T | 0.00 | 0.00 | C |
| RS8843006 | C | 0.20 | 0.36 | T | T | 0.38 | 0.43 | C | C | 0.23 | 0.07 | T | T | 0.50 | 0.43 | C | T | 0.47 | 0.50 | C |
| RS8884972 | A | 0.08 | 0.04 | C | A | 0.04 | 0.19 | C | A | 0.15 | 0.16 | C | A | 0.08 | 0.08 | C | A | 0.12 | 0.14 | C |
| RS8928516 | I | 0.00 | 0.00 | D | I | 0.00 | 0.00 | D | I | 0.00 | 0.00 | D | I | 0.00 | 0.00 | D | I | 0.00 | 0.00 | D |
| RS8955053 | I | 0.07 | 0.00 | D | I | 0.10 | 0.02 | D | I | 0.02 | 0.02 | D | I | 0.22 | 0.14 | D | I | 0.03 | 0.03 | D |
| RS8955054 | A | 0.00 | 0.00 | T | A | 0.00 | 0.00 | T | A | 0.00 | 0.00 | T | *A | 0.05 | 0.03 | T | A | 0.00 | 0.00 | T |
| RS8955055 | I | 0.07 | 0.00 | D | I | 0.08 | 0.02 | D | I | 0.00 | 0.02 | D | I | 0.36 | 0.29 | D | I | 0.00 | 0.02 | D |
| RS9006559 | D | 0.00 | 0.00 | I | D | 0.00 | 0.00 | I | D | 0.00 | 0.00 | I | D | 0.00 | 0.00 | I | D | 0.00 | 0.00 | I |
| RS9013694 | A | 0.20 | 0.29 | G | A | 0.12 | 0.17 | G | A | 0.02 | 0.02 | G | *A | 0.15 | 0.13 | G | A | 0.34 | 0.40 | G |
| RS9044450 | T | 0.00 | 0.00 | C | T | 0.00 | 0.00 | C | T | 0.00 | 0.00 | C | T | 0.06 | 0.02 | C | T | 0.00 | 0.00 | C |
| RS9183439 | A | 0.00 | 0.00 | C | A | 0.00 | 0.00 | C | A | 0.00 | 0.00 | C | A | 0.00 | 0.00 | C | A | 0.00 | 0.00 | C |

*: Failed QC therefore excluded form allelic association analyses. Minor alleles are shown for each breed as they can vary between breeds, e.g. RS24537168 minor allele is ‘T’ in most breeds but is ‘A’ in the Border terrier.
